# Establishing H Angle Hard Tissue and H Angle Soft Tissue Norms in Skeletal Class I Malay Adult Females and the Correlation between the H Angles and Visual Perception of Laypersons

**DOI:** 10.1055/s-0044-1787005

**Published:** 2024-07-16

**Authors:** Joo Ming Cheong, Nur Adlina A. Halim, Norsyamimi Mohammad, Mangaiyarkarasi Sivagnanam, Mohamad Shafiq Mohd Ibrahim

**Affiliations:** 1 Department of Orthodontics, Kulliyyah of Dentistry, International Islamic University Malaysia, Kuantan, Pahang, Malaysia; 2 Dental Polyclinic, Kulliyyah of Dentistry, International Islamic University Malaysia, Kuantan, Pahang, Malaysia; 3 Department of Paediatric Dentistry and Dental Public Health, Kulliyyah of Dentistry, International Islamic University Malaysia, Kuantan, Pahang, Malaysia

**Keywords:** orthodontics, visual perception, Holdaway, angle, esthetics

## Abstract

**Objectives** This study aimed to establish the cephalometric norms of H angle soft tissue and H angle hard tissue of Malaysian Malay adult females, and to evaluate the correlation between H angles and visual perception in skeletal Class I Malay adult females.

**Materials and Methods** Eighty-five lateral cephalograms of skeletal Class I (mean ANB value = 3.15 ± 0.77) Malaysian Malay female patients aged 20 to 40 years (mean age = 28.6 ± 5.86 degrees) taken from October 2017 to December 2021 were measured for H angle soft tissue and H angle hard tissue. Twenty silhouettes were then converted from cephalometric films and were rated according to facial convexity/concavity by 20 laypersons, with re-evaluation after 2 weeks for intra- and interexaminer reliabilities.

**Results** The means of the H angle soft tissue and H angle hard tissue were 15.75 ± 4.16 degrees and 11.64 ± 4.71 degrees, respectively. The intraexaminer reliability test for visual perception ranged from −0.89 to 0.99 indicating poor to excellent reliability, whereas the interexaminer reliability test was 0.82 indicating good reliability. A highly statistically significant association between the H angle soft tissue and H angle hard tissue ( *r* = 0.938; *p* < 0.01) was found. There was no correlation between H angles and visual perception.

**Conclusion** The cephalometric norms of H angle hard tissue and H angle soft tissue in the Malaysian Malay female population were established: 11.64 degrees (±4.71 degrees) and 15.75 degrees (±4.16 degrees), respectively. There was a strong correlation between H angle soft tissue and H angle hard tissue among skeletal Class I Malay adult females. There was no correlation between H angles and the visual perception of laypersons.

## Introduction

Salzmann (1966) 1 was the first to categorize the skeletal pattern into three types, which were Class I, Class II, and Class III. He stated that the profile for skeletal Class I is straight and is the most pleasing. However, the convex profile in skeletal Class II and concave profile in skeletal Class III may detract from ideal facial esthetics. Besides improving function, the desire to seek better facial esthetics was one of the main reasons for patients seeking orthodontic treatment. 2 Therefore, orthodontists need to consider a patient's esthetic value to achieve a desirable orthodontic outcome.

Apart from the dentoskeletal framework, much information could also be gathered from the soft tissue structures covering the hard tissues. 3 Soft tissue plays an important role in the facial relationship as it affects the esthetic outcome. Various angles and soft tissue cephalometrics have been developed to evaluate facial profiles such as Z angle, 4 S line, 5 E line, 6 nasolabial angle, 7 and lower lip to E plane. 8 However, there was no definitive judgment on which assessment was superior to the others in giving the better diagnosis. Holdaway (1983) 9 proposed that the H angle was to be used for evaluation of the patient's profile and comprised H angle hard tissue and H angle soft tissue. H angle hard tissue is the angle formed by the NB line (nasion to point B) and the H line (line tangent to upper lip and soft tissue pogonion), while the H angle soft tissue is the angle formed by the N′PG′ line (soft tissue nasion to soft tissue pogonion) and the H line. H angle is an important measurement adopted in orthodontics to evaluate facial profile characteristics across the upper, middle, and lower thirds. Additionally, it assesses the maxillomandibular relationship, specifically quantifying mandibular protrusion or retrusion. The analysis allows orthodontists to enhance their treatment planning leading to improved facial balance and harmony. According to Holdaway (1983), the normal H angle value is 10 degrees when the convexity measurement is 0 mm.

Various methods have been used to assess facial esthetics which include three-dimensional computer imaging, 10 photometry, 11 anthropometry, 12 and cephalometry. 13 Cephalometric analysis is an objective measurement, as the analysis is performed by identifying the radiographic landmarks through manual or digital tracing to measure the linear and angular values. However, human perception regarding ideal facial esthetic is subjective. The visual perception can vary between individuals which can result in dissimilar treatment expectations between orthodontists and the patients. Hence, choosing appropriate objective measurements (cephalometric variables) that correlate with subjective measurements (visual perception) can help in better diagnosis and treatment planning. In this respect, the H angle was selected as an objective and scientific cephalometric measurement to explore its relationship with subjective visual perception to better understand facial traits and patients' soft tissue characteristics.

Cephalometric norms were developed as a guide to identify the etiology of malocclusion, ultimately leading to correct diagnosis and treatment planning for orthodontic patients. Björk (1947) 14 and Downs (1948) 15 were among the pioneers who established cephalometric standards. However, the classical cephalometric measurements proposed for these analyses were mainly based on Caucasians samples. Most cephalometric studies varied with factors such as ethnicity, gender, and age difference. 16 17 18 Therefore, the cephalometric norms used for one ethnic group may not be applicable as a reference for other ethnic groups due to anthropometric differences. The cephalometric for Malaysian norms were reported in a few studies. 19 20 21 Mohammad et al (2011) 19 obtained cephalometric norms for Malaysian Malays using the Steiner analysis and found that all the linear measurements differed from that of Caucasians. It was apparent that the Malaysian Malays had more protrusive upper and lower lips as compared with Caucasians. Purmal et al (2013) 20 established the cephalometric norms of Malaysian Chinese and Malaysian Indian. However, most local cephalometric studies focused on skeletal and dental cephalometric variables, while soft tissue cephalometric norms were limited in reference. At present, there are no established cephalometric norms for H angles among the Malaysian Malay population. For many years, diagnosis and treatment planning have been based on H angle norms of other racial groups, but this angle could be different for Malaysian Malays.

Therefore, this study aimed to compare the correlation of H angle soft tissue and H angle hard tissue with the visual perception of skeletal Class I Malay female patients in Malaysia as well as to establish the cephalometric norms of H angles for Malaysian Malays.

## Materials and Methods

### Study Design

This was a retrospective cross-sectional study with the convenience sampling method used involving pretreatment lateral cephalometric radiographs.

### Sample Size and Subjects

The sample size was calculated with the single mean formula using the equation as follows:

*n =* ( *Ζ* × σ/∆) ^2^ ,

where *n* = sample size, *Z* = 1.96 for 95% confidence interval (CI) when α = 0.05, σ = standard deviation (SD) of H angle hard tissue = 2.82 (Lersinghanart et al, 2020), 22 Δ = precision (in this study, precision was set at 0.6).

Based on the previous study by Lersinghanart et al (2020), the mean (SD) of H angle hard tissue was 11.13 (±2.82). If the true mean lies within 0.6 degrees angle with 95% CI, a minimum sample size of 85 lateral cephalometric radiographs was required.

For selecting layperson samples, sample size calculation for intraclass correlation coefficient (ICC) was used using the sample size calculator web (Arifin, 2024) 23 with minimum acceptable reliability (ICC) = 0.6, α = 0.05, power of study = 80%, number of repetitions per subject ( *k* ) = 2, and expected dropout rate = 10%. Therefore, the total sample size of included laypersons was 20.

Eighty-five preexisting lateral cephalometric radiographs of Malaysian Malay adult females aged between 20 and 40 years (mean age of 28.6 ± 5.86) with Class I skeletal pattern were retrieved from October 2017 to December 2021 from two private dental clinics in Kuantan city, Pahang state of Malaysia (Dr Fatain's Dental Clinic Taman Tas; Dr Fatain's Dental Clinic Indera Mahkota 3). Patients primarily sought orthodontic treatment to enhance their facial appearance and smile esthetics. The inclusion and exclusion criteria were as follows.

### Inclusion Criteria

1. Nongrowing adult Malay females aged 20 to 40 years.
2. Both parents of each subject were of Malay ethnic origin without any interracial marriages for at least two generations. The ethnicity information was previously verified by C.J.M. via a demographic form during the subject's initial consultation visit.
3. Class I skeletal pattern (ANB within the range of 1–5 degrees). 24
4. Complete records of pretreatment lateral cephalometric radiographs with adequate resolutions and quality (Grade A: diagnostically acceptable) for proper identification based on “Guidance Notes for Dental Practitioners on the Safe Use of X-ray Equipment, 2nd Edition, 2020” by the Faculty of General Dental Practice UK.
5. Radiographs were taken in natural head posture with the Frankfort horizontal plane parallel to the floor. Teeth were in maximum intercuspation, with lips in relaxed position. Cephalogram was taken from the right side of the patient's head at 5 feet from the mid-sagittal plane.
6. Radiographs were taken from the same operator by using the same X-ray machine (72 kV, 10.0 mA, 40 milliseconds) following the manufacturer recommendations of the cephalostat.

The inclusion criteria of laypersons for visual perception of facial profiles were subjects:

1. Aged 20 to 29 years.
2. With no dental knowledge.

### Exclusion Criteria

1. Subjects who had previous orthodontic or orthognathic treatment.
2. Subjects who had a previous history of major maxillofacial surgery.
3. Subjects presented with any developmental deformities, craniofacial anomalies, or any systemic medical conditions that might affect their physical growth.
4. Subjects who had symptoms related to temporomandibular joint disorder.
5. Subjects who had a previous history of any types of prosthetic treatment or major conservative treatment.
6. Records of pretreatment lateral cephalometric radiographs with Grade N (diagnostically not acceptable) resolutions and quality. This included distorted radiographs, or insufficiently clear radiographs which made landmark identifications difficult.

The exclusion criteria of laypersons for visual perception of facial profiles were subjects with visual impairment that affects judgment of visual perception.

### Cephalometric Analysis

The lateral cephalometric films were hand traced and analyzed by a single examiner (N.A.A.H.) on a clear sheet of acetate paper using a 0.5-mm 2B pencil in a darkened room with a light viewing box, a protractor, and a metal ruler. Calibration was done with an orthodontist (C.J.M.) with 7 years of experience to achieve agreement in terms of landmark identifications.

Two weeks after the initial measurements of the H angle soft tissue and H angle hard tissue, 10 randomly chosen cephalometric films were repeated to evaluate for intraexaminer reliability using ICC.

### Visual Perception of Facial Profile

**Fig. 1:**
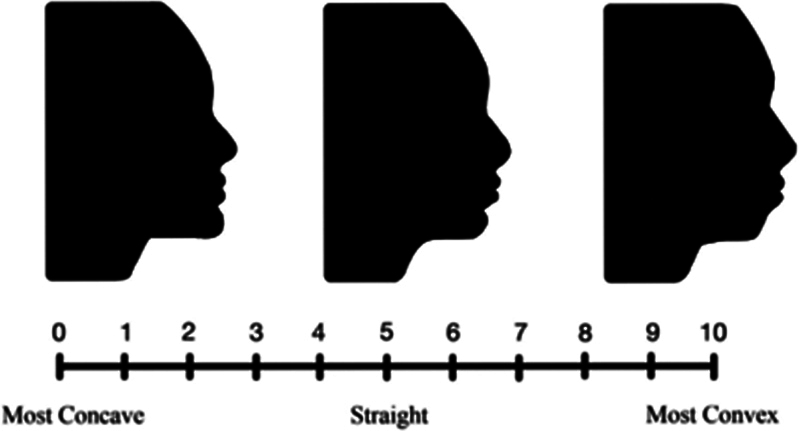
Visual analog scale showing facial silhouette from the most concave (score: 0) to the most convex (score: 10) soft tissue profile.

The outline of the soft tissue profile of 20 randomly selected lateral cephalometric radiographs were traced separately on a clean white background and were made as a silhouette by using the Sketchbook application in iPad eighth generation (Foxconn, Taiwan). The profile silhouette size was standardized at 30 mm (width) × 40 mm (height) with a resolution of 300dpi. A visual analog scale with a score of 0 to 10 was used to rate the convexity and concavity of each soft tissue profile, with 0 indicative of the most concave profile, 5 as straight profile, and 10 as the most convex profile ( Fig. 1 ). The visual perception of soft tissue profiles was distributed to 20 laypersons aged 20 to 29 years (mean age 23 ± 0) with no dental knowledge. Written consent was obtained from them prior to assessment. Two weeks after their first evaluation, the same sets of silhouettes were distributed again to the same 20 laypersons to repeat the facial profile assessments for both intra- and interexaminer reliabilities using ICC.

### Data Analysis

Data analysis was performed using IBM SPSS software version 25.0 (Chicago, Illinois, United States). The normality of data distribution was determined by Kolmogorov–Smirnov's test. Descriptive statistics were used to summarize the demographic backgrounds of the subjects and to obtain the means and SDs of the ANB angle, H angle hard tissue, and H angle soft tissue. ICC was used for intra- and interexaminer reliabilities. The correlation between the H angle soft tissue, H angle hard tissue, and visual perception was evaluated with *p* -value set at <0.05.

## Results

The mean and SD of the measurements are given in Table 1 . The H angle soft tissue and H angle hard tissue had means of 15.75 ± 4.16 degrees and 11.64 ± 4.71 degrees, respectively. The ICC scores for the H angle soft tissue and H angle hard tissue were 0.905 and 0.955, respectively with *p* < 0.01, indicating a high degree of reliability. The intraexaminer reliability test for visual perception ranged from 0.07 to 0.99 indicating poor to excellent reliability, and with one negative value of −0.89 outside theoretically possible range, while the interexaminer reliability test was 0.82, indicating good reliability.

**Table 1:** Means and standard deviation of ANB angle, H angle soft tissue, and H angle hard tissue in Class I Malaysian Malay females

Variables	Mean	Standard deviation
ANB angle (deg)	3.15	0.77
H angle soft tissue (deg)	15.75	4.16
H angle hard tissue (deg)	11.64	4.71

The H angle hard tissue and H angle soft tissue were normally distributed, whereas data for visual perception were not normally distributed. Hence, Pearson's correlation coefficient was used to determine the correlation between H angle hard tissue and H angle soft tissue. The correlation between H angle hard tissue, H angle soft tissue, and visual perception is given in Table 2 . From Pearson's correlation coefficient tests, statistically significant strong and positive correlation were found between the H angle soft tissue and H angle hard tissue ( *r* = 0.938; *p* < 0.05). However, there was no correlation between visual perception and H angle hard tissue, as well as between visual perception and H angle soft tissue as there was no linear relationship between H angles and visual perception.

**Table 2:** Correlation between H angle hard tissue, H angle soft tissue, and visual perception

	H angle soft tissue (deg)	H angle hard tissue (deg)	Visual perception
H angle soft tissue (deg)	1	0.938 a b	Nil
H angle hard tissue (deg)	0.938 a b	1	Nil
Visual perception	Nil	Nil	1

Note: Nil means statistically not significant.

a *p* -Value was set at less than 0.05 to indicate statistical significance.

b Pearson's correlation coefficient.

## Discussion

This retrospective study was designed to establish the H angle hard tissue and H angle soft tissue norms in skeletal Class I adult females and to assess the correlation between the H angles and visual perception. Our study provided cephalometric norms of H angle measurements in skeletal Class I Malaysian Malay females and revealed a significant positive correlation between H angle soft tissue and H angle hard tissue. H angle was investigated in the current study as it is one of the cephalometric parameters in assessing facial profile features, specifically the prominence of the upper lip or prognathism/retrognathism of the soft tissue chin. The study specifically focused on the female Malay population as the highest number of adults seeking orthodontic treatment were females. 25 Dentofacial esthetics were the main concern leading to a more active demand for treatment among females, especially in private orthodontic settings.

The mean ANB value for skeletal Class I Malay female subjects in this study was 3.15 ± 0.77 degrees. Our mean value was higher than the norms of skeletal Class I in Caucasians (2 degrees) 24 and was also inconsistent with a study by Mohammad et al (2011) (mean ANB value = 2.5 ± 1.58 degrees). 19 The disagreement between the studies could be explained by the fact that patients in the study by Mohammad et al (2011) were selected based on Class I incisor classification of British Standards Institute (1983). The use of patients with Class I incisor relationship may not necessarily reflect the underlying ANB value as ANB portrays the relationship of the mandible to maxilla, while the Class I incisor relationship portrays the relationship of the upper and lower incisors to each other. According to Kim et al (2014), 26 skeletal Class III patients may exhibit dentoalveolar compensation to achieve Class I incisor relationship to camouflage the underlying skeletal discrepancy, hence the lower ANB value found in the study by Mohammad et al (2011) compared with ours.

The mean value for H angle soft tissue among Class I Malaysian Malay females in our study was 15.75 degrees. This finding was in agreement with Lersinghanart et al (2020), 22 ALBarakati and Bindayel (2012), 27 and Panezai et al (2021) 28 who reported that the mean values of the H angles were 15.27, 15.16, and 15.05 degrees, respectively. However, our mean H angle value was different with Choki et al (2021) 29 and Noviaranny et al (2022), 30 with a mean value of 20 and 10 degrees, respectively. H angle soft tissue is influenced by the thickness and position of the lips and the nose. Thick upper lip corresponds to a larger H angle soft tissue and since the Malay population mostly have thicker upper lips, the H angle soft tissue is therefore larger compared with the Caucasians. 30 A study by Cezairli (2017) 31 on the comparisons of soft tissue thickness measurements in adult patients with various vertical patterns found that subjects with prominent soft tissue pogonion have a decreased H angle soft tissue value. Another study by Sahin Sağlam and Gazilerli (2001) 32 in children found that an increase in upper lip thickness increased the convexity of soft tissue profile, hence resulting in increased H angle. Thus, it is expected that different soft tissue thickness gives off a variety of mean values in H angle soft tissue in different ethnicities.

In this study, the mean value for H angle hard tissue was 11.64 degrees which was in agreement with Lersinghanart et al (2020) 22 at 11.13 degrees, but different from the finding by Choki et al (2021) 29 at 16.39 degrees, although Thailand females were the subjects in both studies. Lersinghanart et al (2020) recruited skeletal Class I Thailand female patients with a mean ANB value of 3.39 degrees which was similar to the mean ANB value in our study. However, skeletal Class II female Thailand patients with a mean ANB value of 7.27 degrees were included in the study by Choki et al (2021). Therefore, skeletal Class I female Thailand patients have similar H angle hard tissue with skeletal Class I female Malaysian Malay patients.

In this study, H angle soft tissue has a very strong correlation with H angle hard tissue, and this concurred with the result by Choki et al (2021). 29 It was claimed that certain soft tissue structures have a tight association with hard tissue. 33 For instance, the vertical changes of the upper lip could be due to a horizontal change in prosthion (the most inferior anterior point on the maxillary alveolar process between the central incisors) and the position of upper incisors. However, soft tissue can also be affected by its own length, thickness, and functional aspects such as tissue tension.

This study selected laypersons with no dental knowledge to perform visual perception assessment of facial convexity/concavity. The result from this study showed that there was no correlation between visual perception and the H angles. Furthermore, the wide range of intraexaminer reliability, including one negative value, suggests that the judgments on facial profile of the same layperson tend to be variable and subjective at different occasions, although the same profile feature was evaluated. Our result contrasts with that of Lersinghanart et al (2020) 22 and Choki et al (2021) 29 who reported good correlation between H angles and visual perception. These differences can be explained in part by the difference in our study sample being recruited, in which laypersons were selected instead of orthodontic postgraduate students 22 and orthodontic specialists 29 who had orthodontic knowledge. Laypersons were chosen in our study to reflect their visual perception of facial convexity/concavity in real clinical scenarios. In the past, the main aim of orthodontic treatment was to correct dentoalveolar structures based solely on dentition until recently when the soft tissue paradigm shift became the key factor for facial attractiveness following orthodontic treatment. 34 Therefore, it is important to consider the patients' expectations during treatment planning. Patients' views on their ideal profile perception might be different from orthodontists' views; hence in future research, the use of these data from laypersons could be compared with the data from visual perception of orthodontists to ascertain whether there is any difference between them. Communication between orthodontist and patient would be fundamental to bridge the gap in the treatment expectations in order to achieve patient satisfaction of their treatment outcome.

Our study utilized black facial profile silhouettes against white backgrounds to eliminate biases related to skin tone, hairstyle, facial cosmetics, eye color, and hair color. The concern regarding photograph quality such as clarity, color hue, and lighting could therefore be avoided. However, the findings of this study were limited to skeletal Class I patients. Further studies could be performed to determine the association between H angles and visual perception of the skeletal Classes II and III patients, with a particular focus on Class III patients due to their higher prevalence of this malocclusion in Malaysia.

## Conclusion

- The cephalometric norms of the H angle hard and H angle soft tissue in the Malaysian Malay female population were established: H angle hard tissue 11.64 degrees (±4.71 degrees) and H angle soft tissue 15.75 degrees (±4.16 degrees).
- H angle soft tissue and H angle hard tissue were highly correlated in skeletal Class I Malay adult females.
- However, there is no agreement between the H angles and visual perceptions of laypersons. It is recommended that orthodontists should formulate a treatment plan that is customized to individual patient needs, thereby ensuring both patients' expectations and orthodontists' goals on what is considered an ideal facial profile are successfully met.
